# Visual Detection and Evaluation of Latent and Lytic Gene Expression during Epstein-Barr Virus Infection Using One-Step Reverse Transcription Loop-Mediated Isothermal Amplification

**DOI:** 10.3390/ijms141223922

**Published:** 2013-12-09

**Authors:** Xiaoying Liu, Jingfeng Tang, Man Wang, Qiang Ma, Yefu Wang

**Affiliations:** 1 The State Key Laboratory of Virology, College of Life Sciences, Wuhan University, Wuhan 430072, Hubei, China; E-Mails: xiaoyingliu@whu.edu.cn (X.L.); jingfeng@whu.edu.cn (J.T.); maanwong@whu.edu.cn (M.W.); 2 The State Key Laboratory of Respiratory Disease, First Affiliated Hospital of Guangzhou Medical University, Guangzhou 510120, Guangdong, China; E-Mail: mat008@163.com

**Keywords:** EBV, latent/lytic transcripts, RT-LAMP, visual detection

## Abstract

Epstein-Barr virus (EBV)-associated disease exhibits distinct gene expression patterns characterized by the transcription of EBV nuclear antigen (EBNA) 1, EBNA2, latent membrane protein (LMP) 1, LMP2A, and BZLF1 (Zebra). A series of visual reverse transcript loop-mediated isothermal amplification (RT-LAMP) assays were performed to examine the expression of EBNA1, EBNA2, LMP1, LMP2A and BZLF1. The sensitivity of RT-LAMP for these transcripts was approximately equivalent to real-time RT-PCR (RT-qPCR), which was developed to quantify relative levels of EBV transcripts, and 10 to 100-fold more sensitive than conventional RT-PCR. Cross-reactions to other viruses were not observed upon examination of cell lines infected with herpes simplex viruses-1 and -2 (HSV-1 and -2), varicella zoster virus (VZV), human cytomegalovirus (HCMV) or Kaposi’s sarcoma-associated herpesvirus. When applied to 146 specimens, RT-LAMP exhibited high clinical sensitivity and specificity, with an excellent agreement (κ > 0.92) compared to RT-qPCR. These assays are convenient for rapid early diagnosis and for surveillance of EBV-infected individuals by evaluating the EBV transcriptional profile, because the results can be visualized with the naked eye. These assays may be employed in further investigations because they can aid the design of improved therapeutic regimens and can be used specifically in resource-poor settings.

## 1. Introduction

Epstein-Barr virus (EBV) infects more than 95% of people and can lead to life-long, latent infection by attacking the host immune system in immunocompetent individuals [1]. Primary EBV infection usually remains asymptomatic in young children and displays as acute infectious mononucleosis (IM) in 30%–50% of affected adolescents. Young adults tend to recover without sequelae [2,3]. Although IM is generally self-limiting, it occasionally causes serious complications such as airway obstruction, hepatosplenomegaly, hemophagocytic syndrome, pneumonitis and liver damage [4]. Latent EBV infection is involved in the multistep pathogenesis of many malignancies, including lymphoid neoplasms (Hodgkin’s lymphoma (HL), Burkitt’s lymphoma (BL), EBV-associated post-transplantation lymphoproliferative disorders (PTLD), senile EBV-associated B-cell lymphoproliferative disorder and T/NK-cell lymphomas) and epithelial carcinomas (nasopharyngeal carcinoma (NPC), gastric and oral cancers) [5–7]. At least four basic programs (including types I, II, III and 0) of virus latency can been identified on the basis of differential gene expression patterns in different EBV-associated disorders. Only EBV nuclear antigen (EBNA) 1 is expressed in the type I latency program, which is associated with BL [8]. NPC, HL and NK/T cell lymphoproliferative diseases exhibit the type II latency program, characterized by the additional co-expression of latent membrane proteins (LMP1, LMP-2A and LMP-2B) [9]. The latency type III program is frequently found in EBV-associated PTLD, acute infectious mononucleosis (IM) and X-linked lymphoproliferative syndrome (XLP), in which the full spectrum of latent genes (*EBNA1*, *EBNA2*, *EBNA3A*, *EBNA3B*, *EBNA3C*, *EBNA-LP*, *LMP1*, *LMP2A* and *LMP2B*) are expressed [10]. A majority of infected memory B cells only express LMP2A (also considered a type 0 latency), and many of these appear in asymptomatic EBV carriers [11]. Latency can be disrupted by a variety of cellular activators, resulting in the expression of BZLF1 (immediate early gene, Zta or Zebra) associated with the lytic cycle, which induces the switch from latency to lytic replication [12]. Thus, as a lytic infection marker, BZLF1 can be used to distinguish between a latent and a lytic infection [13].

The acute form of EBV-associated diseases, including IM and XLP, and severe complications arising from the infection may result in fatal malignant diseases and can be life-threatening [10]. Early identification of the expression pattern of EBV latent and lytic genes in patients with acute EBV infection would reduce morbidity and mortality [14]. Thus, developing a rapid, reliable and highly sensitive diagnostic method is critical to effectively monitor EBV infection and to understand EBV transcriptional profiles in newly infected cells [15]. Nevertheless, the traditional diagnosis of EBV infection is based on clinical manifestations and serology, which are associated with low sensitivity, frequent false-positives and time-consuming problems; otherwise, some diagnosis such as a serological assay requires convalescent-phase serum that effectively excludes its use for early diagnosis. Real-time PCR (qPCR) is a dramatic improvement, as it is a more sensitive and reproducible assay for the management of viral diseases [16–18]; however, it is instrumentally and technically demanding, compared to a one-step RT-LAMP method. Hence, real-time RT-PCR (RT-qPCR) assays are often unavailable for routine clinical diagnosis in remote rural areas without sufficient medical facilities.

In this study, we reported a technique termed loop-mediated isothermal amplification (LAMP) to resolve the problems that low sensitivity, false-positives, or the requirements of expensive equipment and skilled operation [19]. On the principle of strand-displacing DNA synthesis by the Bst DNA polymerase, the LAMP technique has high specificity, using a set of specially designed primers (two outer primers (F3 and B3), a forward inner primer (FIP, F1c + F2), and a reverse inner primer (BIP, B1c + B2)) that recognize six distinct regions of the target sequence [19]. Amplification from four primers leads to the accumulation of large amounts of products of various lengths, making it more sensitive than conventional RT-PCR [19]. The thermostable reverse transcription (RT) and LAMP reactions can be performed simultaneously, using the same isothermal conditions, at a range of 60–65 °C without sophisticated apparatus, and one-step RT-LAMP can be completed within 1 h or less [20]. LAMP results can be interpreted with inspection by the naked eye by examining either turbidity or fluorescence, and the products can be readily identified by the white magnesium pyrophosphate precipitate generated during the reaction or by adding calcein to the reaction mixture prior to amplification to irradiate fluorescence [21]. Additionally, EBV latent genes are either absent or expressed at infrequent and low levels in infected lymphocytes [7]; the RT-LAMP method can be used to detect the infrequent gene expression of EBV with the intrinsic characteristics of high specificity and sensitivity. Therefore, the technique is feasible for use in resource-poor locations. The rapid detection of EBV DNA by LAMP has been developed by Iwata *et al*., and Nie *et al*. [22,23], and RT-LAMP has been increasingly applied to rapidly diagnose infection by microbial pathogens, such as *Fusarium graminearum* in contaminated wheat seeds, as well as the metastasis of gastric and lung cancers [24–26].

The one-step RT-LAMP assay is able to determine the prevalence rates of EBV infection and evaluate the expression profiles of EBV latent and lytic genes. This assay can be used to identify the status of virus-host interaction in patients with EBV infection thereby providing information for effective therapy. To evaluate the usefulness of this method, we assessed EBV transcriptional patterns using peripheral blood mononuclear cells (PBMCs) from immunocompetent subjects with a primary EBV infection, a past EBV infection, or no EBV infection and compared the results of the RT-LAMP method with RT-qPCR and conventional RT-PCR results.

## 2. Results and Discussion

### 2.1. Establishment of a One-Step RT-LAMP Assay

The one-step RT-LAMP took approximately 60 min (60 min for the RT reaction and the LAMP reaction simultaneously) to perform. Conventional RT-PCR required 2 h (30 min for the reverse transcription (RT) reaction, 70 min for the amplification, and 20 min for agarose gel electrophoresis), and RT-qPCR required approximately 90 min without agarose gel electrophoresis analysis. The calcein can be added into the tube before the reaction, thus results can be visually assessed without opening the tube, accelerating the reaction time and eliminating time-consuming post-amplification operations, as well as significantly reducing multiplex manipulation error and risk of cross-contamination [21,24]. In addition, the only equipment needed for RT-LAMP is a cost-effective laboratory water bath or a heat block that supplies a constant temperature of 63 °C.

**Figure 1.**
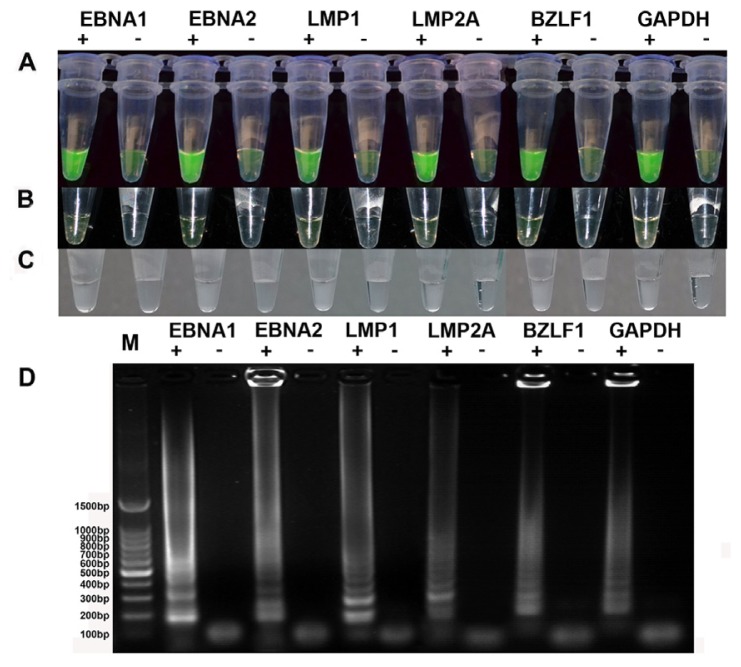
Analysis of RT-LAMP reaction products after amplification. M, 100 bp DNA ladder; +, positive; −, negative. Visual detection was performed using the color change from brown to fluorescent green under UV light. (**A**) A reaction was considered positive if fluorescent calcein dye was detected; (**B**) If the color changed from brown to green in normal light; (**C**) Or if turbidity from the white precipitate was observed; and (**D**) When analyzed by agarose gel electrophoresis, positive RT-LAMP products exhibited a ladder-like pattern.

A one-step RT-LAMP assay was standardized for the rapid detection of EBV latent/lytic transcript (EBNA1, EBNA2, LMP1, LMP2A or BZLF1) and performed using five designed primers (GenBank accession numbers M12553.1, K03333.1, AF023171.1, GU979730.1 and EU340368.1) that recognized a total of six distinct regions in the target sequence (Table 1). Glyceraldehyde-3 phosphate dehydrogenase (GAPDH) is crucial for monitoring the quality control of RNA templates extracted from human samples, and the RT-LAMP primers for GAPDH were designed based on GenBank accession number BC023632.2. The products of the RT-LAMP amplification were examined with three methods: agarose gel analysis, naked-eye visualization and visual fluorescence. When a sample tube contains the target sequence, a white magnesium pyrophosphate precipitate is produced as the reaction progresses, whereas the negative tubes will be transparent and can be visually identified (Figure 1C). Visual fluorescence can be assessed by adding calcein, which yields a clear-cut difference in the reaction mixtures. Positive samples exhibited bright green fluorescence from a brown and green color under ultraviolet (UV) light (302 nm). The color was also changed from brown to green under normal light in positive reactions. All of the detection results for latent transcripts (EBNA1, EBNA2, LMP1, LMP2A) were obtained using the constitutively expressing Raji cell line, and those for the lytic transcript (BZLF1) were obtained from the phorbol 12 myristate 13-acetate (PMA or TPA)-activated Raji cell line under UV light (Figure 1A) and normal light (Figure 1B) [27]. A successful LAMP reaction with species-specific primers produced many bands of different sizes, indicated by a typical ladder-like pattern on a 2% agarose gel, whereas no bands were obtained from negative control reactions (Figure 1D). The above results indicated that visual fluorescence by color change with the addition of calcein dye was more obvious than only naked-eye visualization and more convenient than agarose gel analysis (Figure 1).

### 2.2. Specificity of the RT-LAMP Assay

**Figure 2.**
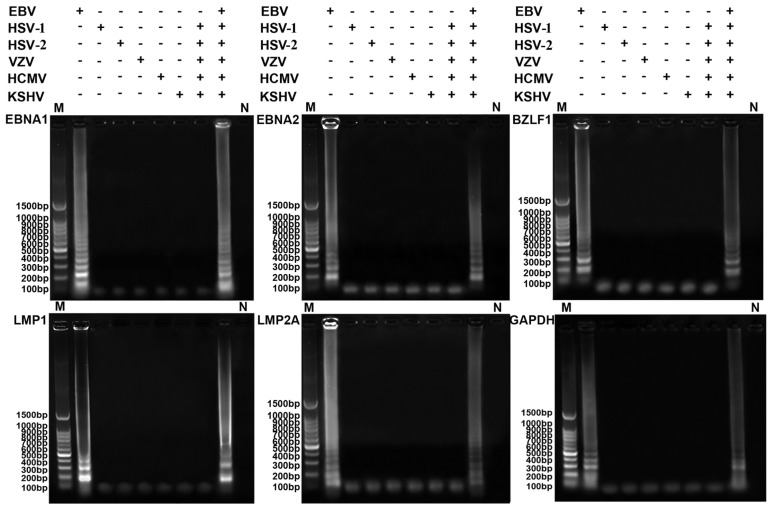
Specificity analysis of RT-LAMP for EBV latent and lytic transcripts (EBNA1, EBNA2, LMP1, LMP2A, BZLF1) and GAPDH. M, 100 bp DNA ladder; +, the reaction with the virus; −, the reaction without the virus; N, negative control. Positive RT-LAMP reactions showed a ladder-like pattern after 2% agarose gel electrophoresis.

Agarose gel electrophoresis analysis showed that a characteristic ladder of multiple bands of different sizes was seen when the templates contained EBV, even when many other kinds of virus (herpes simplex viruses-1 and -2 (HSV-1 and -2), varicella zoster virus (VZV), human cytomegalovirus (HCMV) or Kaposi’s sarcoma-associated herpes virus) were mixed in one sample, and no false positive reactions were observed from other herpes viruses in the absence of EBV (Figure 2). These results indicated that the specific primers for latent and lytic transcripts only amplified the respective target sequence, and there was no cross-reaction with other herpes viruses. These data demonstrate that the established RT-LAMP assays of EBV latent and lytic transcripts have high specificity for detecting the target RNAs.

### 2.3. Comparison of Sensitivity between RT-LAMP, RT-qPCR and Conventional RT-PCR

The sensitivity for each latent and lyitc gene was evaluated using known numbers of EBV-positive cells. A serial dilution of EBV-positive Raji RNA was spiked into RNA from EBV-negative BJAB cells. RNA isolated from cell mixtures containing 1 × 10^6^ EBV-negative BJAB cells and 10-fold serial dilutions of EBV-positive Raji cells ranging from 10^6^ to 10^0^ cells, untreated or treated with TPA, were used for RT-LAMP, RT-qPCR and conventional RT-PCR.

**Figure 3.**
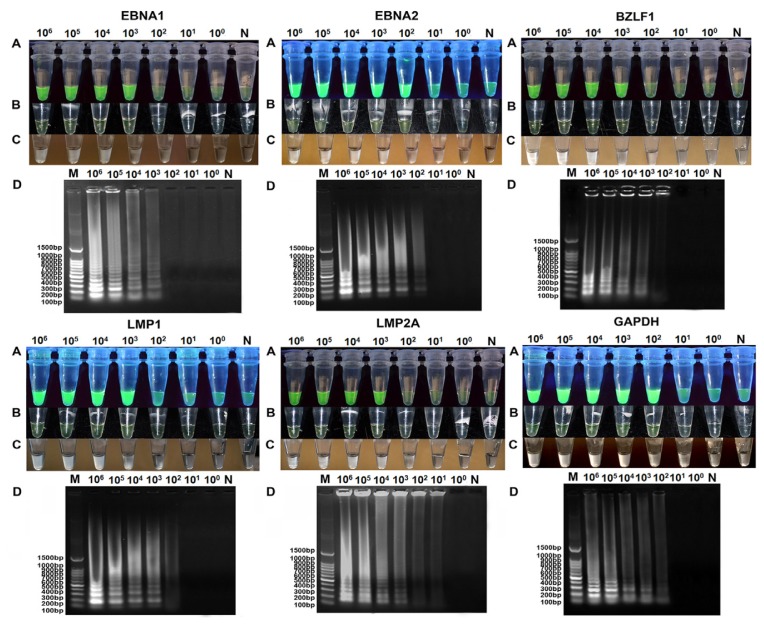
Detection limits of RT-LAMP assays for the evaluation of EBV latent transcripts (EBNA1, EBNA2, LMP1 and LMP2A), a lytic transcript (BZLF1) and GAPDH. The analytical sensitivity of RT-LAMP was determined using cell mixtures containing 1 × 10^6^ BJAB cells and 10-fold serial dilutions of Raji cells ranging from 10^6^ to 10^0^ cells, untreated or treated with TPA. M, 100 bp DNA ladder; N, negative control; 10^6^–10^0^, 10^6^ to 10^0^ cells out of 1 × 10^6^ BJAB cells. The positive results were detected visually in the following ways: (**A**) Observing a color change from brown to green under UV light; (**B**) Observing a color change from brown to fluorescent green under normal light; (**C**) Observing the turbidity of white precipitate generated by the RT-LAMP reaction; and (**D**) Or a ladder-like pattern on a 2% agarose gel.

The detection limits of RT-LAMP by visual inspection using calcein were 10^3^, 10^2^, 10^3^, 10^3^, 10^3^ and 10^2^ Raji cells out of 1 × 10^6^ BJAB cells, respectively, for EBNA1, EBNA2, LMP1, LMP2A, BZLF1 and GAPDH (Figure 3A,B). This was equivalent to the sensitivity of RT-qPCR, but 10 to 100-fold higher than conventional RT-PCR. RT-LAMP assessed by the naked eye without calcein was inferior to electrophoresis analysis and visual fluorescence, although it was more sensitive than conventional RT-PCR (Figure 3C). Agarose gel electrophoresis analysis was slightly more sensitive than detection of the white precipitate inspection with the naked eye, roughly corresponding to the calcein stain (Figure 3D). This finding indicated that the visual fluorescence method for LAMP analysis could be used instead of agarose gel electrophoresis. Adding calcein to the RT-LAMP reaction solution increased the rate of recognition, improving the detection limit to the level of gel analysis, which will facilitate the rapid screening of samples without requiring gel electrophoresis. Only the visual fluorescence assay was performed in further experiments.

**Figure 4.**
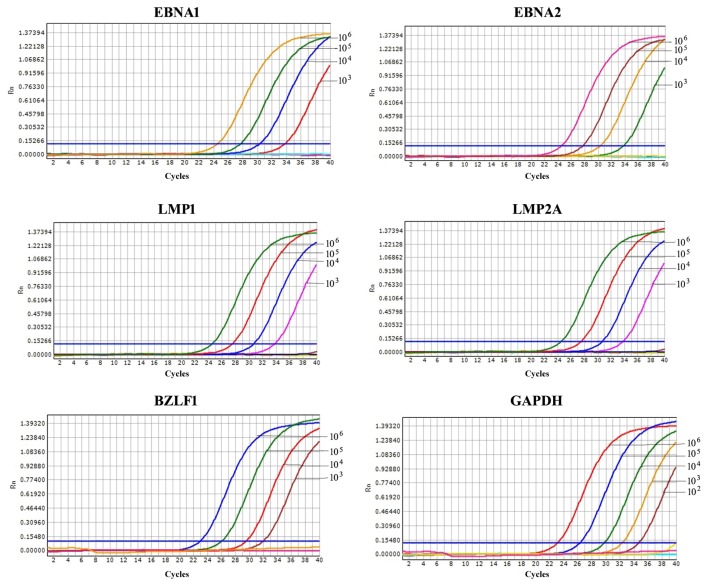
Sensitivity of RT-qPCR for the evaluation of EBV latent transcripts (EBNA1, EBNA2, LMP1 and LMP2A), a lytic transcript (BZLF1) and GAPDH. The sensitivity of RT-qPCR was determined using cell mixtures containing 1 × 10^6^ BJAB cells and 10-fold serial dilutions of Raji cells ranging from 10^6^ to 10^0^ cells, untreated or treated with TPA. 10^6^–10^0^: 10^6^ to 10^0^ Raji cells out of 1 × 10^6^ BJAB cells. The detection limits were 10^3^, 10^3^, 10^3^, 10^3^, 10^3^, and 10^2^ Raji cells out of 1 × 10^6^ BJAB cells for EBNA1, EBNA2, LMP1, LMP2A, BZLF1 and GAPDH, respectively.

**Figure 5.**
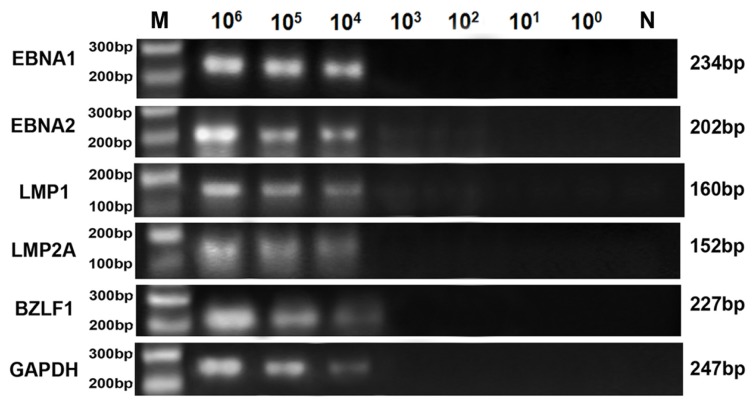
Sensitivity of conventional RT-PCR for the evaluation of EBV latent and lytic transcripts (EBNA1, EBNA2, LMP1, LMP2A and BZLF1) and GAPDH using cell mixtures containing 1 × 10^6^ BJAB cells and 10-fold serial dilutions of Raji cells ranging from 10^6^ to 10^0^ cells, untreated or treated with TPA. M: 100 bp DNA ladder, N: negative control, 10^6^–10^0^, 10^6^ to 10^0^ Raji cells out of 1 × 10^6^ BJAB cells. For all sensitivity assays for conventional RT-PCR using serial 10-fold dilutions of untreated or treated Raji cells, the product observed was the expected band size by 2% gel electrophoresis.

The detection limits of RT-qPCR for EBNA1, EBNA2, LMP1, LMP2A, BZLF1 and GAPDH were 10^3^, 10^3^, 10^3^, 10^3^, 10^3^ and 10^2^ Raji cells out of 1 × 10^6^ BJAB cells, respectively (Figure 4), lower than that of conventional RT-PCR (were all 10^4^ Raji cells out of 1 × 10^6^ BJAB cells) (Figure 5). The products of conventional RT-PCR were verified by sequencing and were in agreement with the predicted sizes of 234, 202, 160, 152, 227 and 247 bp for EBNA1, EBNA2, LMP1, LMP2A, BZLF1 and GAPDH, respectively.

The details of the detection limits of RT-LAMP, RT-qPCR and conventional RT-PCR applied to three positive samples are summarized in Table 2. The detection limit of RT-LAMP for latent and lytic transcripts by visual fluorescence exhibited a lower sensitivity for the positive samples (10^3^, 10^2^, 10^3^, 10^4^ and 10^4^ PBMC out of 1 × 10^6^ BJAB cells) compared to the Raji cell line dilutions (10^3^, 10^2^, 10^3^, 10^3^ and 10^3^ Raji cells out of 1 × 10^6^ BJAB cells). The detection limit was approximately 10-fold lower for the BZLF1 lytic transcript and all the latent transcripts except for LMP1, which was similar for both testing methods. RT-qPCR showed a similar detection limit to RT-LAMP for the PBMC samples, which were 10^3^, 10^3^, 10^3^, 10^3^ and 10^4^ PBMC out of 1 × 10^6^ BJAB cells for EBNA1, EBNA2, LMP1, LMP2A and BZLF1, respectively. Nevertheless, using conventional RT-PCR, the detection limit was 10^4^ PBMC out of 1 × 10^6^ BJAB cells for EBNA1 and LMP1 latent transcripts and 10^5^ PBMC out of 1 × 10^6^ BJAB cells for EBNA2, LMP2A and BZLF1 transcripts, which was 10 to 100-fold less sensitive than the RT-LAMP and RT-qPCR methods.

RT-LAMP was capable of detecting concentrations as low as 0.01% EBV-positive cells, which was sufficient to detect the expression of EBNA2; a concentration of 0.1% of the infected cells showed detectable expression of EBNA1 and LMP1; and a concentration of 1% of the infected cells had detectable LMP2A and BZLF1 transcripts in clinical samples. When detecting samples, several factors should be considered regarding latent and lytic transcript inhomogeneity and the ratio of EBV-infected cells. For instance, target transcript levels are extremely low in the total RNA or PBMC from the positive samples, which can prevent detection of the infection. In healthy carriers, the viral load is stable over time at approximately 1–50 EBV-infected cells per 10^6^ circulating B-cells (0.001%–0.01% infected cells) [9]. EBV-infected B-cells increase to 1%–20% of circulating B-cells when the symptoms of acute IM are apparent, 2–7 weeks after the primary infection [28]. Considering that the detectable level of EBV latent transcripts was approximately 0.01% or even lower level in latently infected lymphocytes, methods with low sensitivity would be ineffective for diagnosis [16]. Thus, our RT-LAMP assay provided a significant improvement for the diagnosis of EBV latent/lytic transcripts and was suitable for identifying EBV gene expression in PBMC from patients with acute primary or past infection.

### 2.4. Application of RT-LAMP Assays for the Surveillance of EBV Gene Expression

Using the RT-LAMP, RT-qPCR and conventional RT-PCR methods, the EBV transcriptional profiles of 146 potentially EBV-positive patients were analyzed. All patients were in the acute phase of febrile illness that was initially suspected to be a primary EBV infection. The results of the evaluation in EBV primary and past infection patients are shown in Table 3. With regard to the latent gene expression of EBNA1, EBNA2, LMP1 and LMP2A, the latent EBNA1 and EBNA2 transcripts were detected in two of the fifty-one subjects (2/51, 3.9%), the LMP1 transcript was detected in eight subjects (8/51, 15.7%) and the LMP2A transcript was detected in eleven subjects (11/51, 21.6%) by RT-LAMP in patients with primary EBV infection. The corresponding values for RT-qPCR (3.9%, 3.9%, 17.6% and 23.5%) were slightly higher than RT-LAMP, and both of these methods were significantly higher than conventional RT-PCR. With respect to lytic gene expression, twenty-one subjects (21/51, 41.2%) were positive by RT-LAMP, and 45.1% of samples were positive by RT-qPCR, whereas only 37.3% were deemed positive by conventional RT-PCR. On the other hand, only EBNA1 and LMP2A were detected, and EBNA2, LMP1 and BZLF1 were absent in patients with past EBV infection. The detection rates of EBNA1 and LMP2A-positive patients by RT-LAMP (56.8% and 62.2%) were slightly lower than RT-qPCR (59.5% and 64.9%), although both exceeded the rate of conventional RT-PCR. As expected, of the 58 subjects not infected with EBV and the healthy blood donors, none were positive for latent or lytic EBV transcript. Employing the RT-qPCR data set as the standard, the RT-LAMP method showed clinical sensitivity values of 100%, 100%, 88.9%, 91.7% and 91.3% for EBNA1, EBNA2, LMP1, LMP2A and BZLF1 in patients with primary EBV infection, and in patients with past EBV infection, it exhibited clinical sensitivity values of 95.5% and 95.8% for EBNA1 and LMP2A, respectively. In the studied samples, the specificity was 100%, and the absolute agreement was higher than 97% with excellent agreement (κ > 0.92) (Table 3). For conventional RT-PCR, the clinical sensitivity values were 100%, 100%, 66.7%, 75% and 73.9% for EBV transcripts in patients with primary EBV infection and 72.7% and 70.8% for EBNA1 and LMP2A in patients with past EBV infection compared to the RT-qPCR data set. The specificity was 100% in the studied samples except for the 92.9% value for BZLF1 in primary EBV infection patients, and the average absolute agreement was 91.1% with good agreement (κ > 0.63). After statistical analysis of viral gene expression, RT-LMAP had a high level of agreement with RT-qPCR and was superior to conventional RT-PCR. The RT-LAMP method was useful for the determination of EBV gene expression in PBMC samples.

EBV-infected naïve tonsillar B cells undergo proliferation (also referred to as the growth program), traverse the germinal center (protected from apoptosis by the default program), and differentiate into memory cells (expressing the latency program) [29]. The stage of EBV-infected B cells can be distinguished by the expression pattern of EBV latent and lytic genes [30]. The infected B cell may present one of three different states during the interaction of EBV with B lymphocytes. The first is a real latent state (only expressing the LMP2A transcript), and the immune response maintains the asymptomatic viral carrier state. The second is an immortalized or persistently low-producing state (referred to as the default program), often accompanied by a full spectrum of latent proteins (LMP1 and EBNA1, -2, -3A, -3B, -3C, and -LP), which signals to the infected B cells to differentiate into latent memory B cells and maintains a persistent infection via the germinal center [31]. The third is a virus-producing phase, encoding approximately 100 viral proteins, including BZLF1, and initiating virus production [10]. The proliferation of EBV-infected lymphocytes can become life-threatening or develop into proliferating lymphoblasts. BZLF1 is detectable during the lytic proliferation of infected B cells [12]. LMP2A is consistently detected in latently infected resting B cells *in vivo*, which induces B-cell survival [32]. EBNA1 is indispensable for the maintenance of the viral genome in dividing immortalized B cells. The expression of EBNA1, LMP-1 and LMP2A is likely involved in the progression of diverse human malignancies caused by EBV [7], suggesting a potential carcinogenesis risk from the infected-cells expressing these latent genes. The results obtained from testing the patients with primary EBV infection showed that all the latent transcripts were detected in infected patients, although there was only one case (Table S1). This expression pattern indicates the maintenance of constant proliferation by EBV during viral latency in newly infected B cells of patients. Another sample had detectable latent and lytic transcripts (Table S1), suggesting that BZLF1 expression during latent infection may be a remnant of lytic infection [12], which is consistent with previous work indicating that a small number of EBV-positive cells expressing BZLF1 can be detected in latently infected proliferating cells [5]. The low frequency of EBNA1 and EBNA2 in patients with primary infection may contribute to the immunological escape mechanism of EBV primary infection. The lytic gene *BZLF1* was detectable with a high positive rate during primary EBV infection but not in the 37 subjects with past EBV infection, indicating the possibility of producing large quantities of virons (lytic state). The high frequency of EBNA1 and LMP2A in patients with past infection might be involved in the mechanism suppressing EBV lytic reactivation [32,33].

A new latency pattern (with EBNA1 and LMP2A without LMP1 expression) that resembled the phenotypically representative latency I defined for BL lines was exhibited in patients with past EBV infection (Table S2), suggesting that EBV latency patterns may be more complicated in clinical practice [16,34]. Another possible reason for the different latency profile was that the EBV expression pattern in the clinical specimens might be different from *in vitro* cultured cell lines or that our data set should contain a larger number of infected samples. Thus, further efforts are necessary to test the method with a larger variety of patients with EBV-related diseases. A major drawback of RT-LAMP is that it is not quantitative as the RT-qPCR assay is, but because the purpose of this research was to diagnose the prevalence of EBV infection and determine the expression patterns of EBV-infected cells, and the RT-LAMP assay had a sensitivity equal to RT-qPCR, quantitation was not necessary. Recently, Rafailidis *et al*., found that using antiviral agents to treat severe EBV infection in immunocompetent patients may be beneficial by avoiding lytic virus reactivation in patients with acute severe primary EBV infection with ensuing immunosuppression [35].

## 3. Experimental Section

### 3.1. Clinical Specimens

Whole blood samples from 146 patients were collected in the First Affiliated Hospital of Guangzhou Medical College from September 2011 to November 2012 and tested by ELISA. The subjects were classified into three groups: Group 1 (*n* = 51) included patients with primary EBV infection diagnosed upon typical serological findings (anti-VCA IgM and/or IgG seropositive and anti-EBNA Ig variable depending upon the time from the onset of the infection); group 2 (*n* = 37) included patients with past EBV infection (anti-VCA IgG seropositive and IgM seronegative, anti-EBNA Ig seropositive); and group 3 (*n* = 58) included 58 healthy blood donors without EBV infection as negative controls (anti-VCA and anti-EBNA Ig seronegative) [36].

### 3.2. Cell Lines

The EBV-positive B cell line Raji (ATCC, CCL-86) constitutively expresses a subset of latent viral genes consisting of six nuclear antigens (EBNA1, EBNA2, EBNA3A, EBNA3B, EBNA3C, and EBNA-LP), three latent membrane proteins (LMP1, LMP2A, and 2B), and two small nonpolyadenylated nuclear RNAs (EBV-encoded RNA 1 (EBER1) and EBER2) [17]. The RT-LAMP positive control for EBV latent transcripts was assessed using RNA extracted from the Raji cell line, whereas the EBV lytic transcript was assessed using RNA obtained from the Raji cell line after activation with 20 ng/mL TPA (Sigma, St. Louis, MO, USA) [27]. RNA from the EBV-negative B cell line BJAB was used as a negative control [17]. All B cell lines (the untreated Raji cell line, the Raji cell line treated with TPA, and the BJAB cell line) were maintained in RPMI 1640 medium (GIBCO, Grand Island, NY, USA) supplemented with 10% heat-inactivated fetal bovine serum (FBS) (GIBCO, Grand Island, NY, USA), 100 units/mL penicillin and 100 μg/mL streptomycin in a humidified incubator (Thermo Scientific, Cornelius, OR, USA) at 37 °C and 5% CO_2_. Cells were collected at a density of 1 × 10^6^ cells, and then centrifuged at 500× *g* for 5 min. The cell pellets were stored at −80 °C until use. Additionally, human cord blood, Vero cells (ATCC CCL-81) infected with HSV-1, Vero cells (ATCC CCL-81) infected with HSV-2, MRC-5 cells (ATCC CCL-171) infected with VZV, Mink lung cells (ATCC PTA-3450) infected with HCMV, and BCP-1 cells (ATCC CRL-2294) infected with Kaposi’s sarcoma-associated herpesvirus.

### 3.3. PBMC Isolation and Total RNA Preparation

PBMC were isolated from EDTA-venous blood by centrifugation over a Ficoll-Hypaque density gradient at 1500 rpm for 30 min at room temperature, washed in 0.9% NaCl, counted and adjusted to a final concentration of 1 × 10^6^ cells/mL. Total RNA was extracted from previously collected Raji cell pellets and PBMC from clinical specimens using the RNA reagent Total RNA Isolation System (Promega, Madison, WI, USA) according to the manufacturer’s protocols. DNA removal was performed by on-column deoxyribonuclease digestion for 15 min at room temperature using the RNaseFree DNase Set (Qiagen, Hilden, Germany). Then, the extracted RNA was measured using spectrophotometry and purity was ascertained at an OD_260_/OD_280_ ratio between 1.8 and 2.0. Total RNA was eluted with 50 μL DEPC-treated water and tested immediately or stored at −80 °C until used.

### 3.4. RT-LAMP Reaction

Based on the mRNA sequences of EBV latent (*EBNA1*, *EBNA2*, *LMP1*, *LMP2A*) and lytic (*BZLF1*) genes, a set of four primers (two outer primers (B3, F3) and two inner primers (BIP, FIP)) for each RT-LAMP assay were automatically designed using Primer Explorer V3 software (http://primerexplorer.jp/elamp3.0.0/index.htmL; Eiken Chemical, Tokyo, Japan). To quantify and prove the integrity of isolated RNA, RT-LAMP for glyceraldehyde-3 phosphate dehydrogenase (GAPDH) was also carried out. RT-LAMP primers targeting GAPDH were designed using the same software.

The one-step RT-LAMP assays for EBV latent and lytic transcripts were performed separately in a 25-μL reaction mixture consisting of 2.5 μL Bst DNA polymerase buffer (10× buffer, consisted of 20 mM Tris-HCl, 10 mM KCl, 2 mM MgSO_4_, 10 mM (NH_4_)_2_SO_4_ and 0.1% Triton X-100); 3 μL dNTPs (10 mM, Takara, Osaka, Japan); 3 μL MgSO_4_ (50 mM); 3.5 μL betaine (5 M); 0.1% Tween-20; 0.5 mol/L MnCl_2_; 8 U Bst DNA polymerase (8 U/μL, New England Biolabs, Ipswich, MA, USA); 5 U AMV reverse transcriptase (5 U/μL, Promega, Madison, WI, USA); 4 U of RNasin; 40 pmol of each inner primer FIP and BIP for *EBNA1*, *EBNA2*, or *LMP1*; 55 pmol each inner primer FIP and BIP for *LMP2A*, *BZLF1* or *GAPDH*; 20 pmol each outer primer F3 and B3 for *EBNA1*, *EBNA2*, or *LMP1*; and 25 pmol each outer primer F3 and B3 for *LMP2A*, *BZLF1* or *GAPDH*. Each reaction was mixed separately and 5 μL total RNA was added for a final total volume of 25 μL. Positive and negative controls were included in each run to ensure the quality of the assay and that no contamination was presented, respectively. The one-step reaction was performed when the isothermal characteristics of RT-LAMP were set at a constant temperature of 63 °C for 60 min.

When the reaction was completed, the white turbidity caused by magnesium pyrophosphate precipitate was visually observed after a pulse spin as a positive result. Visual fluorescence detection was performed following the addition of 1 μL of calcein (1.25 nM) into the reaction tube and incubation at 63 °C. Positive results could be distinguished by a color change under normal light and UV light (302 nm). The amplified products were also detected by agarose gel electrophoresis, followed by staining with ethidium bromide under UV light.

### 3.5. Conventional RT-PCR and RT-qPCR

The primers or probes sequence used in conventional RT-PCR and RT-qPCR reactions for *EBNA2*, *LMP1*, *LMP2A*, *BZLF1* and *GAPDH* were designed (GenBank accession numbers: K03333.1, AF023171.1, GU979791.1, EU340368.1 and BC023632.2), the primers or probes sequence for EBNA1 were designed based on AY825078.1 used in conventional RT-PCR and M13941.1 used in RT-qPCR, which are listed in Table S3.

Viral mRNA expression was amplified by RT-qPCR, as described previously, using the published set of specific primers and probes for the detection of EBNA1, EBNA2, LMP1 and BZLF1 transcripts [17] and were modified according to target sequence variations. The reaction was performed with the One-Step PrimeScript RT-PCR kit (Takara, Osaka, Japan) using an Applied Biosystems 7500 Fast Real-Time PCR System (ABI, Foster, CA, USA) in a 20 μL mixture containing 2 μL total RNA; 10 μL 2× One Step RT-PCR buffer III; 0.4 μL PrimeScript RT enzyme mix II; 0.4 μL ExTaq; 5 pmol of each primer (forward primer and reverse primer) and probe for *EBNA1*, *EBNA2*, *LMP1* or *GAPDH*; 6 pmol of each primer and probe for *LMP2A* or *BZLF1*; and DEPC-treated water up to 20 μL. The thermal cycling profile consisted of a 5 min reverse transcription step at 42 °C, followed by 20 s at 95 °C, then 40 cycles of amplification (95 °C for 5 s and 60 °C for 20 s). Fluorescence was recorded at 60 °C.

Conventional RT-PCR was performed in a 50 μL reaction mixture using the One-Step PrimeScript RT-PCR kit (Takara, Osaka, Japan), which contained 2 μL of total RNA; 25 μL of 2× One step buffer; 2 μL of PrimeScript 1 Step Enzyme Mix; 30 pmol of each primer (forward primer and reverse primer) for *EBNA1*, *EBNA2*, *LMP1*, *LMP2A* or *GAPDH*; 20 pmol of each primer for *BZLF1*; 5 μL template RNA and DEPC-treated water up to 50 μL. The thermal cycle conditions were 50°C for 30 min, 2 min at 94 °C, 30 cycles (94 °C for 30 s, 57 °C for 30 s, and 72 °C for 45 s). The products were analyzed by 2% agarose gel electrophoresis.

### 3.6. Specificity of the RT-LAMP Assay

The specificities of the RT-LAMP assays were examined by cross-reactivity tests with five other herpes viruses that are often found co-infected with EBV and are capable of establishing a latent infection, HSV-1 and -2, VZV, HCMV and Kaposi’s sarcoma-associated herpesvirus were evaluated using a mixture of the five listed herpes viruses (contain HSV-1 and -2, VZV, HCMV and Kaposi’s sarcoma-associated herpesvirus) and a mixture also containing EBV (contain HSV I and II, VZV, HCMV, Kaposi’s sarcoma-associated herpesvirus and EBV).

### 3.7. Sensitivity Comparison of the Assays

To compare the sensitivities of the RT-LAMP, RT-qPCR and conventional RT-PCR methods for detecting latent and lytic transcripts, extracts containing 1 × 10^6^ BJAB total RNA and 10-fold serial dilutions of Raji (treated or untreated with TPA) total RNA (RNA isolated from 10^6^ cells to 10^0^ cells out of 1 × 10^6^ BJAB cells) were used.

The sensitivity of these three assays were further confirmed by amplifying the triplicate RNA templates obtained from three positive samples for at least one lytic/latent transcript which were randomly selected from the three subjects groups in a parallel run of control cell dilutions (10^6^ cells to 10^0^ cells out of 1 × 10^6^ BJAB cells). In the presence of RNA extracted from 1 × 10^6^ PBMC, which approximates the RNA extracted from 1 × 10^6^ Raji cells, the total RNA of the positive samples extracted from PBMC was serially 10-fold diluted, ranging from 10^6^ to 10^0^ cells out of 1 × 10^6^ BJAB cells. Each standardized positive dilution was tested in triplicate. To confirm the positive reaction and to compare the sensitivity of RT-LAMP using agarose gel analysis, naked eye visualization or visual fluorescence, the results were analyzed using these three methods.

### 3.8. Application of RT-LAMP in Clinical Specimens and Comparison with RT-qPCR and Conventional RT-PCR

To assess the utility of RT-LAMP compared to RT-qPCR and conventional RT-PCR, total RNA was extracted from all of the clinical samples; subsequently, transcripts were detected in parallel using these three methods. Latent and lytic transcripts from EBV-associated patients were evaluated using these three assays, using the negative and positive control as a reference to identify the gene expression patterns of EBV. The amplification products of RT-LAMP were analyzed by direct visual inspection of the reaction tube under UV light.

The results of RT-qPCR were used to as a reference standard. Clinical sensitivity, specificity, absolute agreement and kappa values (κ values) of the assay were calculated using Cross-Tab in SPSS 17.0 statistical package (SPSS Inc., Chicago, IL, USA). The level of agreement between RT-LAMP and RT-qPCR assays was assessed using the κ coefficient. This test verifies whether agreement exceeds chance levels, with κ ≥ 0.6 indicating good agreement and κ ≥ 0.9 indicating excellent agreement [37].

## 4. Conclusions

In conclusion, our one-step visual RT-LAMP approach for rapidly detecting EBV latent (*EBNA1*, *EBNA2*, *LMP1* and *LMP2A*) and lytic (*BZLF1*) gene expression has been shown to be a reliable, quick and promising system. The RT-LAMP method has a high sensitivity and specificity, similar to RT-qPCR, and the ease of visualization makes it simpler and cost-effective. Our RT-LAMP method is potentially useful for evaluating latent and lytic gene expression during EBV infection, or as a complementary tool used in combination with the routine serology and qPCR used in a reference diagnostic laboratory and resource-poor settings [38].

## Supplementary Information



## Figures and Tables

**Table 1.** LAMP primers used in this present study.

Name	Position a	Primer Sequences (5′→3′)
*LMP1*^b^ F3	944–961	ACCCTAGCGACTCTGCTG
*LMP1* B3	1100–1118	TGGGGGTCGTCATCATCTC
*LMP1* FIP	F1c: 1022–1041	GTCTGTCATCGAAGGCGGGCTTTT c
	F2: 971–989	GAGGCCCTCCAGAATTGAC
*LMP1* BIP	B1c: 1055–1076	ATCCACACCTTCCTACCCTGCTTTTT c
	B2: 1081–1099	CACCGGAACCAGAAGTACC
*LMP2A*^b^ F3	1084–1104	GGCAGTATTTTGCAAACAAAC
*LMP2A* B3	1250–1267	ATGAAGAGTATGCCAGCG
*LMP2A* FIP	F1c: 1158–1182	GCTTGTTTTCTTCAACTAAACAGGATTTT c
	F2: 1106–1125	TCAAGAGTTTAAGCAGCACT
*LMP2A* BIP	B1c: 1185–1209	TTGGATTGTAACACACATTTTACGCTTTT c
	B2: 1226–1249	ACAATCAGTAATAACATGCAGAAC
*EBNA1*^b^ F3	407–426	GAGTAGTCTCAGGGCATCCT
*EBNA1* B3	927–946	ATGTGTCTCCCTTCTCTCCT
*EBNA1* FIP	F1c: 468–489	CGGTGAATCTGCTCCCAGGTCTTTTT c
	F2: 427–444	CTGGAGCCTGACCTGTGA
*EBNA1* BIP	B1c: 492–512	GCGGCCGTCTCCTTTAAGTGTTTTT c
	B2: 904–923	CCATTTCCAGGTCCTGTACC
*EBNA2*^b^ F3	711–730	AATCAACCTGATTCCCCCTG
*EBNA2* B3	919–936	CGCTGGGTGGTTACTGTG
*EBNA2* FIP	F1c: 777–796	AGCACTTACCCGAGCGGGAGTTTT c
	F2: 737–756	CCTCCACTTACAACCAAGCC
*EBNA2* BIP	B1c: 835–856	AGGCAAACCTCCAATCACCAGCTTTT c
	B2: 880–899	TAATGCGTAGCAGCCACTCT
*BZLF1*^b^ F3	6–25	GGACCCAAACTCGACTTCTG
*BZLF1* B3	210–228	AGGAAACCACGACCCAGTT
*BZLF1* FIP	F1c: 94–115	GCCCTCCCAGGTCCTGATAGACTTTT c
	F2: 45–62	TGACCCATACCAGGTGCC
*BZLF1* BIP	B1c: 130–149	TTGCCTTGTGTGCTGTGGCCTTTT c
	B2: 192–209	GGTGCGGCTGAAACATGA
*GAPDH*^b^ F3	248–267	CCATCTTCCAGGAGCGAGAT
*GAPDH* B3	449–468	GCTGATGATCTTGAGGCTGT
*GAPDH* FIP	F1c: 313–333	GGTGAAGACGCCAGTGGACTCTTTT c
	F2: 273–292	CAAAATCAAGTGGGGCGATG
*GAPDH* BIP	B1c: 351–370	GGCTCATTTGCAGGGGGGAGTTTT c
	B2: 411–430	TCACACCCATGACGAACATG

a Nucleotide positions;

b GenBank accession numbers for *LMP1*, *LMP2A*, *EBNA1*, *EBNA2*, *BZLF1* and *GAPDH* are AF023171.1, GU979730.1, M12553.1, K03333.1, EU340368.1, and BC023632.2;

c TTTT is the linker between F1c and F2 in FIP, between B1 and B2c in BIP.

**Table 2.** Sensitivity tests of RT-LAMP, RT-qPCR and conventional RT-PCR methods using PBMC from positive samples.

Assay		PBMC/1 × 10^6^ BJAB cells						

		10^6^	10^5^	10^4^	10^3^	10^2^	10^1^	10^0^
RT-LAMP	EBNA1	+	+	+	+	−	−	−
	EBNA2	+	+	+	+	±(2/3)	−	−
	LMP1	+	+	+	+	−	−	−
	LMP2A	+	+	+	+	−	−	−
	BZLF1	+	+	+	−	−	−	−

RT-qPCR	EBNA1	+	+	+	+	−	−	−
	EBNA2	+	+	+	+	−	−	−
	LMP1	+	+	+	+	−	−	−
	LMP2A	+	+	+	+	−	−	−
	BZLF1	+	+	+	−	−	−	−

Conventional RT-PCR	EBNA1	+	+	+	−	−	−	−
	EBNA2	+	+	−	−	−	−	−
	LMP1	+	+	+	−	−	−	−
	LMP2A	+	+	−	−	−	−	−
	BZLF1	+	+	−	−	−	−	−

+, triplicate assay showed all positive; ±, triplicate assay showed both positive and negative (positive number/test number); −, triplicate assay showed all negative.

**Table 3.** Clinical sensitivity and specificity of RT-LAMP, RT-qPCR and conventional RT-PCR for the five EBV-related genes in patients with EBV primary or past infection and control subjects.

		RT-qPCR a	RT-LAMP					Conventional RT-PCR				
		
Sample	Transcript	No. positive patients (%)	No. positive patients (%)	Clinical sensitivity b	Clinical specificity b	Absolute agreement b	κ value c	No. positive patients (%)	Clinical sensitivity d	Clinical specificity d	Absolute agreement d	κ value e
Group 1 (*n* = 51)	EBNA1	2 (3.9%)	2 (3.9%)	100%	100%	100%	1.00	2 (3.9%)	100%	100%	100%	1.00
	EBNA2	2 (3.9%)	2 (3.9%)	100%	100%	100%	1.00	2 (3.9%)	100%	100%	100%	1.00
	LMP1	9 (17.6%)	8 (15.7%)	88.9%	100%	98%	0.93	6 (11.8%)	66.7%	100%	94.1%	0.77
	LMP2A	12 (23.5%)	11 (21.6%)	91.7%	100%	98%	0.94	9 (17.6%)	75%	100%	94.1%	0.82
	BZLF1	23 (45.1%)	21 (41.2%)	91.3%	100%	98%	0.92	19 (37.3%)	73.9%	92.9%	84.3%	0.68
Group 2 (*n* = 37)	EBNA1	22 (59.5%)	21 (56.8%)	95.5%	100%	97.3%	0.95	16 (43.2%)	72.7%	100%	83.8%	0.68
	EBNA2	0	0	−	−	−	−	0	−	−	−	−
	LMP1	0	0	−	−	−	−	0	−	−	−	−
	LMP2A	24 (64.9%)	23 (62.2%)	95.8%	100%	97.3%	0.94	17 (45.9%)	70.8%	100%	81.1%	0.63
	BZLF1	0	0	−	−	−	−	0	−	−	−	−
Group 3 (*n* = 58)	EBNA1	0	0	−	−	−	−	0	−	−	−	−
	EBNA2	0	0	−	−	−	−	0	−	−	−	−
	LMP1	0	0	−	−	−	−	0	−	−	−	−
	LMP2A	0	0	−	−	−	−	0	−	−	−	−
	BZLF1	0	0	−	−	−	−	0	−	−	−	−

a Diagnostics of samples using RT-qPCR as the standard;

b RT-LAMP comparison with RT-qPCR;

c Agreement of results between RT-LAMP and RT-qPCR;

d Conventional RT-PCR comparison with RT-qPCR;

e Agreement of results between conventional RT-PCR and RT-qPCR.
