# Supplementary material for: Visual Detection and Evaluation of Latent and Lytic Gene Expression during Epstein-Barr Virus Infection Using One-Step Reverse Transcription Loop-Mediated Isothermal Amplification
Source: Int J Mol Sci. 2013 Dec 9;14(12):23922–40. doi: 10.3390/ijms141223922 (PMC3876086; doi:10.3390/ijms141223922)
Supplement: Supplementary file 1 [file ijms-14-23922-s001.pdf]

Supplementary Information

Table S1. EBV latent/lytic gene expression in patients with EBV primary infection by RT-LAMP (group 1).

Subjects	No. (%) of 51 Specimens with Pos Result					
	EBNA1 Lat I/II/III [a]	EBNA2 Lat III [a]	LMP1 lat II/III [a]	LMP2A Lat 0/II/III [a]	BZLF1 IE [b]	GAPDH
1	Neg [c]	Neg	Neg	Neg	Neg	Pos
2	Neg	Neg	Neg	Neg	Neg	Pos
3	Neg	Neg	Neg	Neg	Pos d	Pos
4	Neg	Neg	Neg	Neg	Neg	Pos
5	Neg	Neg	Neg	Neg	Pos	Pos
6	Neg	Neg	Neg	Neg	Pos	Pos
7	Neg	Neg	Neg	Neg	Neg	Pos
8	Pos	Pos	Pos	Pos	Pos	Pos
9	Neg	Neg	Pos	Pos	Neg	Pos
10	Neg	Neg	Neg	Neg	Neg	Pos
11	Neg	Neg	Neg	Neg	Pos	Pos
12	Neg	Neg	Neg	Neg	Neg	Pos
13	Neg	Neg	Pos	Pos	Neg	Pos
14	Neg	Neg	Neg	Neg	Neg	Pos
15	Neg	Neg	Neg	Neg	Neg	Pos
16	Neg	Neg	Neg	Neg	Pos	Pos
17	Neg	Neg	Neg	Neg	Neg	Pos
18	Pos	Pos	Pos	Pos	Neg	Pos
19	Neg	Neg	Pos	Pos	Neg	Pos
20	Neg	Neg	Neg	Neg	Pos	Pos
21	Neg	Neg	Neg	Neg	Neg	Pos
22	Neg	Neg	Neg	Neg	Neg	Pos
23	Neg	Neg	Neg	Neg	Neg	Pos
24	Neg	Neg	Neg	Neg	Pos	Pos
25	Neg	Neg	Neg	Neg	Pos	Pos
26	Neg	Neg	Neg	Pos	Neg	Pos
27	Neg	Neg	Neg	Neg	Pos	Pos
28	Neg	Neg	Neg	Neg	Neg	Pos
29	Neg	Neg	Neg	Neg	Pos	Pos
30	Neg	Neg	Neg	Neg	Pos	Pos
31	Neg	Neg	Neg	Neg	Neg	Pos
32	Neg	Neg	Pos	Pos	Neg	Pos
33	Neg	Neg	Neg	Neg	Pos	Pos
34	Neg	Neg	Neg	Neg	Neg	Pos
35	Neg	Neg	Neg	Neg	Pos	Pos
36	Neg	Neg	Neg	Neg	Neg	Pos
37	Neg	Neg	Neg	Neg	Neg	Pos
38	Neg	Neg	Pos	Pos	Neg	Pos
39	Neg	Neg	Neg	Neg	Pos	Pos
40	Neg	Neg	Neg	Neg	Pos	Pos
41	Neg	Neg	Neg	Neg	Pos	Pos
42	Neg	Neg	Neg	Neg	Neg	Pos
43	Neg	Neg	Pos	Pos	Neg	Pos
44	Neg	Neg	Neg	Pos	Neg	Pos
45	Neg	Neg	Neg	Neg	Neg	Pos
46	Neg	Neg	Neg	Neg	Pos	Pos
47	Neg	Neg	Neg	Neg	Pos	Pos
48	Neg	Neg	Neg	Neg	Pos	Pos
49	Neg	Neg	Neg	Neg	Neg	Pos
50	Neg	Neg	Neg	Neg	Pos	Pos
51	Neg	Neg	Neg	Pos	Neg	Pos

[a] Latent transcript expression; [b] Lytic gene expression; [c] Pos, Positive; Neg, Negative.

Table S2. EBV latent/lytic gene expression in patients with EBV past infection by RT-LAMP (group 2).

Subjects	No. (%) of 37 Specimens with Positive Result					
	EBNA1 Lat I/II/III [a]	EBNA2 Lat III [a]	LMP1 Lat II/III [a]	LMP2A Lat 0/II/III [a]	BZLF1 IE [b]	GAPDH
1	Neg [c]	Neg	Neg	Neg	Neg	Pos
2	Pos	Neg	Neg	Pos d	Neg	Pos
3	Pos	Neg	Neg	Pos	Neg	Pos
4	Neg	Neg	Neg	Pos	Neg	Pos
5	Pos	Neg	Neg	Neg	Neg	Pos
6	Pos	Neg	Neg	Pos	Neg	Pos
7	Neg	Neg	Neg	Neg	Neg	Pos
8	Pos	Neg	Neg	Neg	Neg	Pos
9	Pos	Neg	Neg	Pos	Neg	Pos
10	Pos	Neg	Neg	Neg	Neg	Pos
11	Pos	Neg	Neg	Pos	Neg	Pos
12	Neg	Neg	Neg	Pos	Neg	Pos
13	Neg	Neg	Neg	Neg	Neg	Pos
14	Neg	Neg	Neg	Pos	Neg	Pos
15	Pos	Neg	Neg	Neg	Neg	Pos
16	Neg	Neg	Neg	Pos	Neg	Pos
17	Pos	Neg	Neg	Pos	Neg	Pos
18	Neg	Neg	Neg	Neg	Neg	Pos
19	Neg	Neg	Neg	Pos	Neg	Pos
20	Pos	Neg	Neg	Pos	Neg	Pos
21	Neg	Neg	Neg	Pos	Neg	Pos
22	Pos	Neg	Neg	Neg	Neg	Pos
23	Neg	Neg	Neg	Neg	Neg	Pos
24	Pos	Neg	Neg	Pos	Neg	Pos
25	Pos	Neg	Neg	Neg	Neg	Pos
26	Neg	Neg	Neg	Pos	Neg	Pos
27	Neg	Neg	Neg	Pos	Neg	Pos
28	Pos	Neg	Neg	Neg	Neg	Pos
29	Pos	Neg	Neg	Pos	Neg	Pos
30	Pos	Neg	Neg	Pos	Neg	Pos
31	Neg	Neg	Neg	Neg	Neg	Pos
32	Neg	Neg	Neg	Pos	Neg	Pos
33	Pos	Neg	Neg	Neg	Neg	Pos
34	Pos	Neg	Neg	Pos	Neg	Pos
35	Neg	Neg	Neg	Pos	Neg	Pos
36	Pos	Neg	Neg	Pos	Neg	Pos
37	Pos	Neg	Neg	Pos	Neg	Pos

[a] Latent transcript expression; [b] Lytic gene expression; [c] Pos, Positive; Neg, Negative.

Table S3. Primers and/or probes used for RT-qPCR and conventional RT-PCR.

Method	Primer and Probe	Position [a]	Product Size (bp)	Sequence (5'-3')
Conventional RT-PCR	*LMP1* FP [b]	511–530	160	CTCCTTTGGCTCCTCCTGTT
	LMP1 RP [b]	650–670		GTCTGCCCTCGTTGGAGTTAG
	LMP2A FP	205–226	152	GACCGTCACTCGGACTATCAAC
	LMP2A RP	338–356		CTTCCTCTGCCCGCTTCTT
	EBNA1 FP	170–188	234	GGTTTGGAAAGCATCGTGG
	EBNA1 RP	383–403		CAAAGGGGAGACGACTCAATG
	EBNA2 FP	1873–1892	202	GGGCATGGACCTCTAGCATC
	EBNA2 RP	2055–2074		GGGTAAGCCTCGGTTGTGAC
	BZLF1 FP	273–293	227	ACCTCAGCTGTTCCCAGT CTC
	BZLF1 RP	479–499		ATTCTGGCTGTWGTGGTTTCC [c]
	GAPDH FP	528–548	247	TGGTATCGTGGAAGGACTCAT
	GAPDH RP	755–774		TTCTAGACGGCAGGTCAGGT
RT-qPCR	*LMP1* FP	247–272	98	CTTTGTCTACTCCTACTGATGATCAC
	LMP1 RP	323–344		CCGAAGATGAACAGCACAATTC
	LMP1 Probe	282–307		FAM-CATCGCTCTCTGGAATTTGCA CGGAC-TAMRA
	LMP2A FP	264–282	92	GCAACACGACGGGAATGAC
	LMP2A RP	337–355		TTCCTCTGCCCGCTTCTTC
	LMP2A Probe	299–323		FAM-CCTACTCTCCACGGGATGACT CATC-TAMRA
	EBNA1 FP	270–289	129	GAGCCTGACCTGTGATCGTC
	EBNA1 RP	378–399		TAGGCCATTTCCAGGTCCTGTA
	EBNA1 Probe	333–357		FAM-CGGCCGTCTCCTTTAAGTGTG AATC-TAMRA
	EBNA2 FP	878–900	127	AGAGAGTGGCTGCTACGCATTAG
	EBNA2 RP	983–1004		TCACAAATCACCTGGCTAAGCC
	EBNA2 Probe	921–947		FAM-CAGTAACCACCCAGCGCCAAT CTGTCT-TAMRA
	BZLF1 FP	495–513	88	AGAATCGCTGGAGGAATGC
	BZLF1 RP	563–582		CTTAAACTTGGCCCGGCATT
	BZLF1 Probe	523–547		FAM-CTAGAAATAAAGCGATACAAG AATC-TAMRA
	GAPDH FP	33–51	223	GGTGAAGGTCGGAGTCAAC
	GAPDH RP	236–255		GAAGATGGTGATGGGATTTC
	GAPDH Probe	116–135		FAM-TTGCCATCAATGACCCCTTC-T AMRA

[a] Nucleotide positions; [b] FP (forward primer), RP(reverse primer); [c] W = A/T.

© 2013 by the authors; licensee MDPI, Basel, Switzerland. This article is an open access article distributed under the terms and conditions of the Creative Commons Attribution license (http://creativecommons.org/licenses/by/3.0/).
